# Admission-time immunologic patterns in hospitalized children with *Mycoplasma pneumoniae* pneumonia: a molecular load–antibody titer phenotyping analysis

**DOI:** 10.3389/fped.2026.1814508

**Published:** 2026-07-15

**Authors:** Xianyao Wang, Hachao Zhou, Lingling Jiang, Haipeng Lin, Wenshan Zhong, Ruiling Ma, Zhiwei Xiao, Shaofen Lin, Jing Lin, Wanli Zhuang, Yutao Guo, Mingxiang Lin

**Affiliations:** 1 Department of Pediatrics, Shantou Central Hospital, Shantou, Guangdong, China; 2 Shantou University Medical College, Shantou, Guangdong, China

**Keywords:** antibody titer, load–titer phenotypes, *Mycoplasma pneumoniae* pneumonia, pathogen co-detection, pathogen load

## Abstract

**Background:**

Targeted next-generation sequencing (tNGS) provides a semi-quantitative signal of *Mycoplasma pneumoniae* (MP) molecular burden, whereas antibody titers reflect humoral immune response. However, the joint relationship between MP molecular load, antibody response, and illness timing remains poorly characterized in hospitalized children with MP pneumonia (MPP). We described tNGS-based co-detection profiles in the full cohort and performed admission-time load–titer phenotyping in children with MP-only pneumonia.

**Methods:**

We conducted a retrospective cohort study of hospitalized children with tNGS-confirmed MPP from January to December 2024. Co-detection profiles and associated clinical characteristics were summarized in the full cohort. Formal load–titer phenotyping was restricted to children with MP-only pneumonia to reduce co-detection-related heterogeneity. In this subgroup, two-dimensional kernel density estimation (2D-KDE) was applied to log₁₀-transformed normalized MP reads and log₂(titer + 1)-transformed antibody titers. Onset-to-admission intervals were also summarized across antibody titer categories.

**Results:**

Among 402 children, MP-only, MP + viral co-detection, and MP + bacterial co-detection accounted for 39.3%, 30.1%, and 30.6% of children, respectively. MP + viral co-detection was associated with younger age, longer cough duration, higher white blood cell (WBC) and platelet counts, lower C-reactive protein (CRP), higher antibody titers, and slightly greater resource use, whereas MP + bacterial co-detection generally resembled MP-only. Antibody titers showed a timing-related gradient: children with the first positive titer level of 1:40 were typically admitted around day 6 after illness onset, whereas very high titers corresponded to longer onset-to-admission intervals. Among 158 children with MP-only pneumonia, 2D-KDE suggested three admission-time molecular–serologic patterns: P1 high-load/seronegative, P2 high-load/high-titer, and P3 lower-load/high-titer, accounting for 29.7%, 45.6%, and 24.7% of children, respectively. Across P1–P3, onset-to-admission interval and fever duration increased, the neutrophil-to-lymphocyte (N/L) ratio rose, and platelet counts peaked in P2 before partially declining in P3.

**Conclusions:**

Admission-time molecular load–antibody titer phenotyping in children with MP-only hospitalized MPP suggested three immunologic patterns. The high-load/high-titer pattern indicates that persistent MP molecular signal and established humoral response may coexist at hospitalization. This joint framework may help clinicians contextualize MP tNGS signals in relation to host immune status and illness timing, rather than relying on molecular load or antibody titer alone.

## Introduction

*Mycoplasma pneumoniae* is a leading cause of community-acquired pneumonia in children worldwide and accounts for a substantial proportion of pediatric hospitalizations for lower respiratory tract infection (1–3). Most cases of *M. pneumoniae* pneumonia (MPP) are self-limiting, yet a subset of children experience prolonged fever, extensive pulmonary consolidation, or refractory/severe disease requiring escalated care (4, 5). Early characterization of infection stage and host response is therefore clinically relevant for management decisions and risk assessment.)

Microbiologic diagnosis of MPP traditionally relies on serology and nucleic acid–based tests such as polymerase chain reaction (PCR) (6, 7). Serology is widely available but strongly timing-dependent: Immunoglobulin M (IgM) typically becomes detectable after an initial seronegative window and may persist for months, limiting its ability to distinguish early infection from convalescence (8). Combined IgM/immunoglobulin G (IgG) interpretation and particle agglutination antibody titers can reflect the host humoral immune response, but they do not directly measure the concurrent molecular burden of *M. pneumoniae*. Conversely, nucleic acid–based assays, including targeted next-generation sequencing (tNGS), can detect *M. pneumoniae* and, under standardized workflows, provide semi-quantitative molecular signals based on normalized read counts (9–11). Thus, tNGS-derived MP signal and antibody titers provide complementary pathogen-side and host-side measurements for interpreting the relationship between pathogen burden and humoral immune status.

In clinical practice, this relationship is often interpreted through a simplified temporal framework: high molecular load with absent or low antibody titers during early infection, followed by lower molecular load with high antibody titers after serologic response has developed. However, antibody emergence, antibody persistence, and pathogen clearance are not necessarily synchronous. Molecular and serologic measurements may therefore provide discordant or complementary information at admission, and the true joint distribution of these two measurements in hospitalized children may be more complex than suggested by either marker alone. This uncertainty provides the rationale for examining MP molecular signal and antibody titers within a shared quantitative space.

In real-world tNGS testing, MP is frequently detected together with additional viral or bacterial signals. These co-detections provide important real-world microbiologic context, but they may also introduce biological heterogeneity and complicate the interpretation of normalized MP reads. This is particularly relevant for bacterial detections from upper-airway specimens, which may reflect colonization, background flora, or low-abundance carriage rather than confirmed lower-respiratory bacterial coinfection (12, 13). Therefore, formal load–titer phenotyping is most interpretable in children with MP-only pneumonia, while co-detection profiles are best described as the broader real-world background of the cohort.

Our primary objective was to characterize admission-time molecular load–antibody titer patterns in MP-only MPP using two-dimensional kernel density estimation (2D-KDE). We also described pathogen co-detection profiles and associated clinical characteristics in the full cohort to provide real-world co-detection context for the MP-only phenotyping analysis.

## Materials and methods

### Study design and participants

This retrospective cohort included children aged >1 month to <14 years who were hospitalized with MPP at Shantou Central Hospital, a tertiary care center in Shantou, China. Consecutive admissions from 1 January to 31 December 2024 were screened. The study complied with the Declaration of Helsinki and was approved by the institutional ethics committee of Shantou Central Hospital [Approval No. (2025) 119]. Because only de-identified data were used, informed consent was waived.

#### Eligibility criteria

Eligibility was based on the 2023 evidence-based guideline for pediatric MPP (14). Children were included if they met all of the following criteria:

1. Age >1 month to <14 years;
2. Clinically diagnosed pneumonia based on history, symptoms, physical findings, and radiographic evidence;
3. Detection of *M. pneumoniae* by targeted next-generation sequencing from oropharyngeal swabs collected at admission;
4. Laboratory-reported estimated *M. pneumoniae* concentration >1 × 10⁶ copies/mL.

#### Rationale for the estimated MP concentration threshold

To improve the specificity of the primary analytic cohort for current clinically relevant MP pneumonia with robust molecular signals, we excluded cases with laboratory-reported estimated MP concentrations below 1 × 10⁶ copies/mL. In the original data review, children with MP-only pneumonia below this threshold corresponded to a distinctly low normalized-read range and may include recent prior or resolving infection, residual nucleic acid, sampling variability, or non-causative co-detection rather than current active MP-related pneumonia. Below-threshold children with available antibody titers were included only in the threshold sensitivity analysis, and the results are presented in Supplementary Table S3.

Children were excluded if they met any of the following criteria:

1. Missing *M. pneumoniae*–specific serologic titers at admission;
2. Missing key clinical data, such as age, fever duration, or imaging;
3. Known severe primary immunodeficiency, malignant disease under active chemotherapy, or long-term systemic immunosuppressive therapy;
4. Readmission within one month after a confirmed episode of MPP, with only the first admission retained;
5. Unresolved severe pneumonia from a previous episode;
6. Congenital bronchial malformations.

### Pathogen co-detection grouping and analytical strategy

Based on tNGS results (panel spectrum summarized in Supplementary Table S1), children were first classified according to whether *M. pneumoniae* was detected alone or together with additional viral and/or bacterial pathogens. For the primary analysis, three tNGS-based co-detection categories were defined:

1. MP-only: detection of *M. pneumoniae* without additional viral or bacterial pathogens;
2. MP + viral co-detection: detection of *M. pneumoniae* with at least one viral pathogen, with or without bacterial detections;
3. MP + bacterial co-detection: detection of *M. pneumoniae* with at least one bacterial pathogen and no viral pathogen.

This primary three-category scheme was used to describe the real-world co-detection background and associated clinical characteristics of hospitalized children with tNGS-confirmed MPP, rather than to establish causality or confirm lower-respiratory tract coinfection. Mixed viral–bacterial detections were grouped with MP + viral co-detection in the primary analysis because viral detections were considered more directly relevant to respiratory co-detection patterns, whereas bacterial detections from upper-airway specimens may also reflect colonization or non-causative carriage.

To assess the robustness of this grouping strategy, we additionally performed a four-group sensitivity analysis separating children into MP-only, MP + viral-only co-detection, MP + bacterial-only co-detection, and MP + viral + bacterial co-detection. The four-group sensitivity results are provided in Supplementary Table S2.

Formal load–titer phenotyping was restricted to children with MP-only pneumonia to reduce co-detection-related heterogeneity in normalized MP read interpretation. Co-detection groups were therefore used for descriptive cohort-level comparisons, whereas 2D-KDE modeling of the joint distribution of MP molecular signal and antibody titer was performed in the MP-only subgroup.

### Laboratory methods

#### Specimen collection and tNGS workflow

Oropharyngeal swabs were collected within 24 h of admission using sterile disposable swabs. The posterior pharyngeal wall was swabbed 3–4 times; the swab tip was then immersed in viral transport medium and broken off to ensure complete submersion. Samples were transported promptly to Guangzhou KingMed Diagnostics Group Co., Ltd. (Guangzhou, China) for tNGS testing using a respiratory pathogen tNGS panel (MH75) and the KM MiniSeqDx-CN sequencing platform (Guangzhou KingQiRui Biotechnology Co., Ltd.) according to the manufacturers' procedures.

#### Serologic antibody titers

Serum *M. pneumoniae* antibody titers were measured using a particle agglutination assay (Fujirebio, Tokyo, Japan) according to the manufacturer's instructions. Sera were serially diluted two-fold starting at 1:40 and incubated with antigen-coated gelatin particles at room temperature for 3 h. The highest dilution showing visible agglutination was recorded as the titer. Titers <1:40 were considered seronegative and coded as 0, and titers were categorized into six groups: 0 (negative), 1:40, 1:80, 1:160, 1:320, and >1:320. For joint load–titer analysis, antibody titers were treated as an ordinal measure of serologic response intensity.

#### Semi-quantitative normalization of tNGS read counts

To enable within-cohort comparison of MP signal intensity, raw tNGS reads were converted to normalized read counts defined as MP-mapped reads per 100,000 post–quality-control non-host reads (reads per 100,000; RPK). RPK values were log₁₀-transformed [log₁₀(RPK)] to stabilize variance and reduce the influence of extreme values. Further details on normalization rationale and quality control are provided in Supplementary Methods.

### Clinical data collection and variable definitions

Clinical data were extracted from electronic medical records, including demographics [age, sex, and body mass index (BMI)], clinical features (fever duration before admission, peak temperature, and cough duration at admission), laboratory indices at or near admission [white blood cell (WBC) count, platelet count, neutrophil and lymphocyte percentages, neutrophil-to-lymphocyte (N/L) ratio, C-reactive protein (CRP), lactate dehydrogenase (LDH), and antibody titers], and tNGS results. Radiographic findings were based on chest radiographs. Short-term outcome variables included supplemental oxygen use, oxygen therapy duration, pediatric intensive care unit (PICU) admission, mechanical ventilation, and in-hospital death.

### Statistical analyses

All analyses were performed using SPSS version 26.0 (IBM Corp., Armonk, NY, USA) and GraphPad Prism version 9.0 (GraphPad Software, San Diego, CA, USA). 2D-KDE, peak identification, and phenotype assignment were implemented in Python (version 3.10; NumPy/SciPy) with Matplotlib/Seaborn for visualization.

Continuous variables were assessed for normality using the Shapiro–Wilk test. Normally distributed variables are reported as mean ± standard deviation (SD) and compared using one-way analysis of variance (ANOVA) with Bonferroni *post hoc* tests. Non-normally distributed variables are reported as median [interquartile range (IQR)] and compared using the Kruskal–Wallis test. When Kruskal–Wallis tests were significant, pairwise comparisons were performed using Dunn's test with Bonferroni adjustment; letters following values in tables reflect these adjusted pairwise results. Categorical variables are reported as counts (%) and compared using *χ*² tests or Fisher's exact tests, as appropriate. All tests were two-tailed, with *P* < 0.05 considered statistically significant.

#### 2D-KDE phenotyping in children with MP-only pneumonia

To characterize how pathogen-side molecular signal and host-side antibody response were paired at admission, 2D-KDE was applied to the joint distribution of log₁₀(RPK) and log₂(titer + 1) to characterize the load–titer density surface. Normalized MP reads were log₁₀-transformed because read-count data were right-skewed and spanned several orders of magnitude. Antibody titers were transformed as log₂(titer + 1) because the particle agglutination assay uses serial two-fold dilutions, and the addition of 1 allowed seronegative samples coded as 0 to be included in the transformed scale.

The KDE used a Gaussian kernel with bandwidth selected by Scott's rule. Three local maxima of the KDE surface were identified algorithmically, and their coordinates were defined as the phenotype peak centers (P1–P3). Each child was assigned to the phenotype whose peak center was nearest in Euclidean distance in the [log₁₀(RPK), log₂(titer + 1)] space, with distance computed on the log-transformed coordinates as defined above. This nearest-center rule yields a deterministic classification given the bandwidth rule and peak coordinates. These phenotypes were regarded as data-driven admission-time load–titer patterns rather than predefined clinical stages or treatment categories. Clinical, laboratory, radiographic, and management-related variables were compared across P1–P3 using the methods described above. Low-signal children with MP-only pneumonia below the estimated concentration threshold were not used to define the primary P1–P3 phenotypes. For threshold sensitivity analysis, the same log-transformed and standardized 2D-KDE procedure was repeated after adding low-signal cases with available antibody titers to the primary MP-only cohort. The resulting density structure and exploratory comparisons with the primary P1–P3 groups are presented in Supplementary Table S3.

#### tNGS-based co-detection analyses in the full cohort

For full-cohort co-detection analyses, the primary factor was the three-level tNGS-based pathogen co-detection category: MP-only, MP + viral co-detection, and MP + bacterial co-detection. Continuous variables were compared using Kruskal–Wallis tests or ANOVA, as appropriate, and categorical variables were compared using *χ*² or Fisher's exact tests. These analyses were used to describe cohort-level co-detection profiles and associated clinical characteristics, rather than to infer confirmed lower-respiratory coinfection or causality. Sensitivity analyses using the four-group scheme are provided in Supplementary Table S2. Detailed handling of antibody titers is described in the Supplementary Methods.

## Results

### tNGS-based pathogen co-detection patterns among hospitalized children with MPP

**Figure 1:**
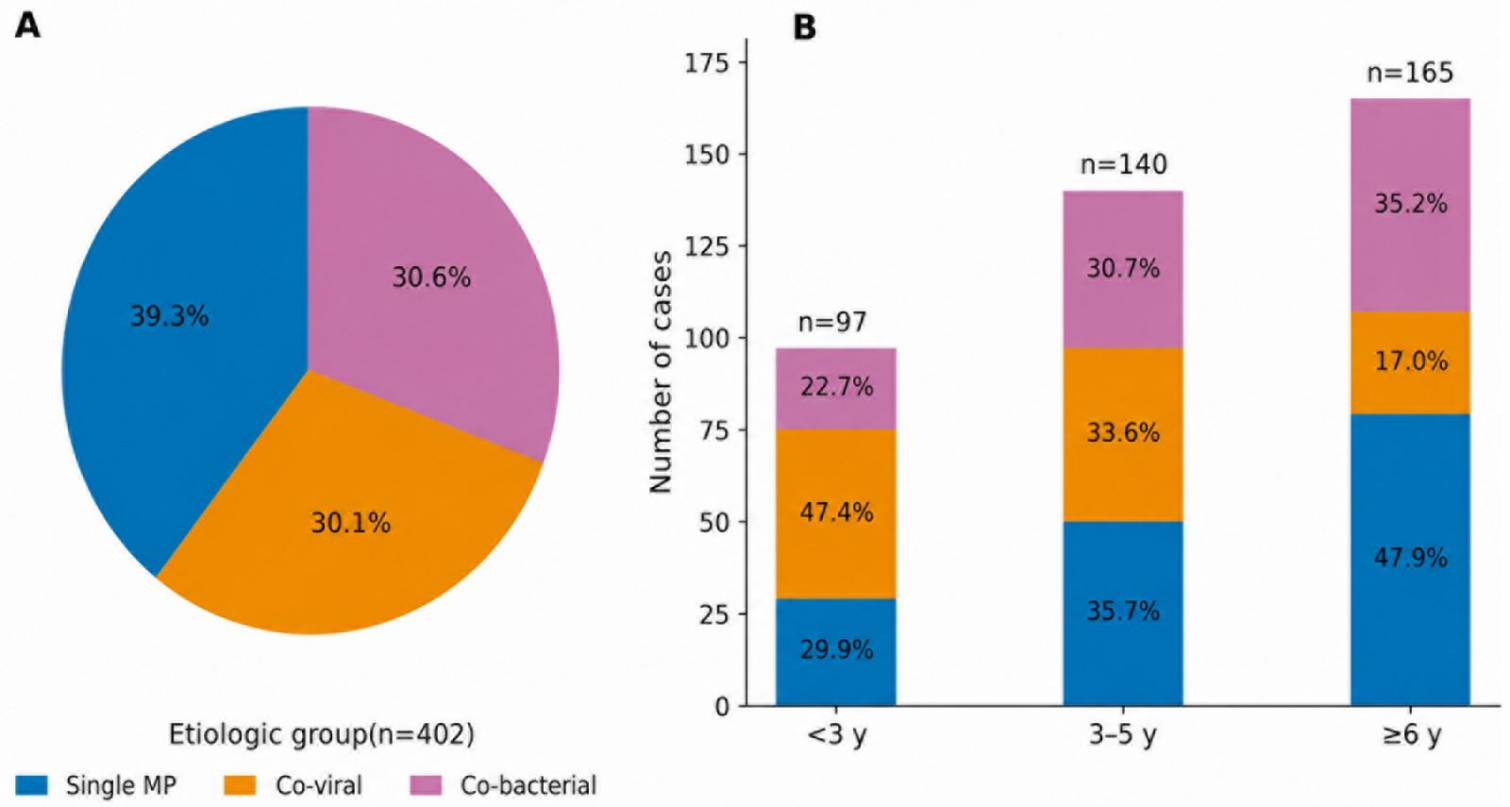
tNGS-based pathogen co-detection patterns and age distribution in 402 hospitalized children with *Mycoplasma pneumoniae* pneumonia. **(A)** Overall distribution of the three tNGS-based pathogen co-detection categories: MP-only, MP + viral co-detection, and MP + bacterial co-detection. **(B)** Age-stratified distribution of the three co-detection categories. Percentages inside each stacked bar indicate the within-age-group proportion of each co-detection category, and n above each bar indicates the number of children in that age group. MP, *Mycoplasma pneumoniae*; tNGS, targeted next-generation sequencing.

After applying the eligibility criteria, 402 children with tNGS-confirmed *M. pneumoniae* infection were included. Among them, 158 children had MP-only pneumonia, 121 had MP + viral co-detection, and 123 had MP + bacterial co-detection, accounting for 39.3%, 30.1%, and 30.6% of the cohort, respectively. Thus, additional viral or bacterial signals were detected in approximately 60% of this real-world hospitalized cohort (Figure 1A). Age distribution differed significantly across co-detection categories: MP + viral co-detection was concentrated in children <3 years, whereas MP-only and MP + bacterial co-detection became more frequent with increasing age (Figure 1B).

### Monthly distribution of hospitalized children with tNGS-confirmed *M. pneumoniae* infection by co-detection category

**Figure 2:**
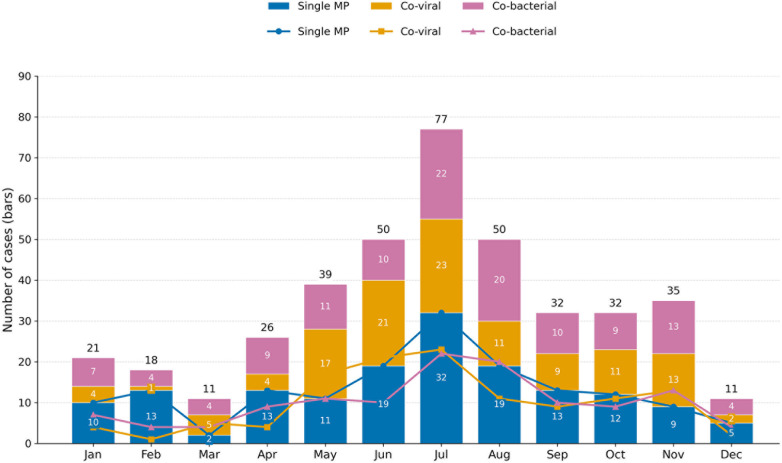
Monthly distribution of hospitalized children with *Mycoplasma pneumoniae* pneumonia by co-detection category.

Children hospitalized with MPP showed a marked seasonal pattern, with a trough in late winter–early spring (February–March) and a peak in July (77 cases, 19.2%). MP-only remained the largest category across most months. MP + viral co-detection increased during the warm-season peak from May to August, whereas MP + bacterial co-detection was more evenly distributed throughout the year (Figure 2).

### Viral detection spectrum among children with MP + viral co-detection

**Figure 3:**
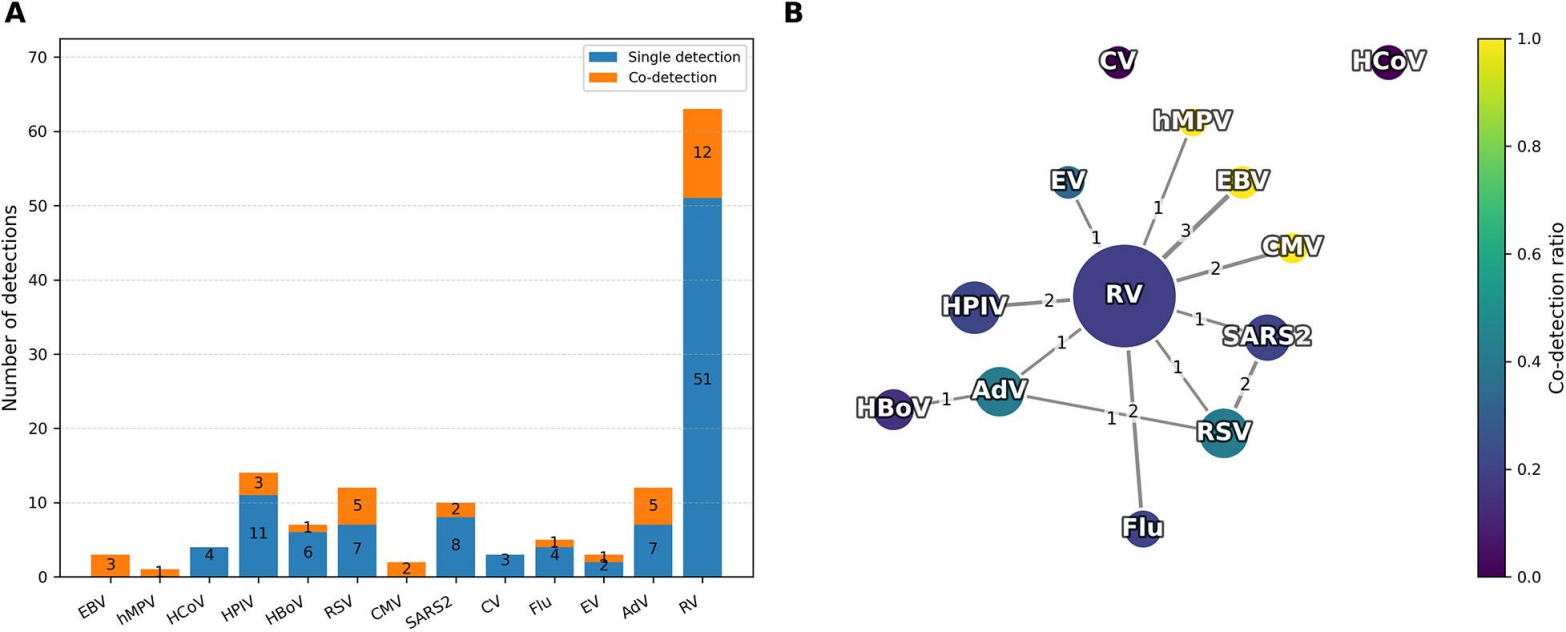
Distribution network of respiratory viruses among children with MP + viral co-detection. **(A)** Counts of viral detections, stratified by single viral detection versus mixed viral co-detection; **(B)** network of pairwise viral co-detection events among MP-positive children. RV, rhinovirus; PIV, parainfluenza virus; RSV, respiratory syncytial virus; AdV, adenovirus; SARS-CoV-2, severe acute respiratory syndrome coronavirus 2; HBoV, human bocavirus; Flu, influenza virus; HCoV, human coronavirus; EV, enterovirus; CV, coxsackievirus; EBV, Epstein–Barr virus; CMV, cytomegalovirus; HMPV, human metapneumovirus.

Among children with MP + viral co-detection (*n* = 121), 139 viral detections were recorded because each virus in mixed viral detections was counted separately (Figure 3A). Rhinovirus (RV) was most common, followed by PIV, RSV, AdV, and SARS-CoV-2; HBoV, Flu, and HCoV were less frequent, and several other viruses (EV, CV, EBV, CMV, HMPV) were only sporadically detected. RV accounted for nearly half of all viral detections and had the highest proportion of mixed viral co-detection, whereas HCoV, Flu, and HBoV were predominantly single viral detections. The co-detection network (Figure 3B) showed that RV formed the broadest set of links with other respiratory viruses, indicating that it participates in diverse viral co-detection combinations in MP-positive children, although most RV detections were not part of mixed viral events.

### Clinical characteristics across tNGS-based pathogen co-detection categories

Clinical characteristics and short-term outcomes across the three tNGS-based pathogen co-detection categories are summarized in Table 1. Compared with MP-only and MP + bacterial co-detection, children in the MP + viral co-detection group were younger and had longer cough duration, whereas fever duration and onset-to-admission interval were broadly comparable. The MP + viral co-detection group also showed a distinct inflammatory profile, with higher WBC and platelet counts but lower CRP. BMI, N/L ratio, and LDH did not differ significantly across groups.

**Table 1:** Descriptive clinical characteristics and short-term outcomes across tNGS-based pathogen co-detection categories.

Variable	MP-only (*n* = 158)	MP + viral co-detection (*n* = 121)	MP + bacterial co-detection (*n* = 123)	*P* value
Demographics				
Age, months	66 (36–96)a	36 (12–60)b	60 (36–84)a	<0.001
Male sex, *n* (%)	87 (55.1)	72 (59.5)	71 (57.7)	0.752
BMI, kg/m²	14.58 (13.80–16.06)	14.79 (13.79–16.01)	14.61 (13.69–15.82)	0.733
Clinical course at admission				
Fever duration, days	5.00 (4.00–7.00)	5.00 (2.00–7.00)	5.00 (3.00–6.50)	0.243
Tmax, °C	39.30 (38.90–39.70)a	39.00 (38.30–39.50)b	39.30 (38.70–39.80)a	0.004
Onset-to-admission interval, days	7.00 (5.00–7.00)	7.00 (5.00–10.00)	6.00 (5.00–8.50)	0.063
Cough duration, days	6.00 (4.00–7.00)a	7.00 (5.00–10.00)b	5.00 (4.00–8.00)a	0.021
Laboratory markers				
WBC, ×10⁹/L	7.71 (6.35–9.25)a	9.07 (6.87–11.16)b	8.21 (6.25–10.59)ab	0.005
N/L ratio	2.13 (1.46–3.23)	1.87 (1.25–2.77)	2.01 (1.51–3.00)	0.214
Platelet count, ×10⁹/L	263.00 (213.25–319.50)a	294.00 (223.00–379.00)b	250.00 (213.00–323.50)a	0.008
CRP, mg/L	13.72 (4.39–24.57)a	8.34 (0.00–19.20)b	12.01 (4.21–25.30)a	0.016
LDH, U/L	324.50 (285.00–391.75)	337.00 (288.00–410.00)	329.00 (277.00–375.50)	0.236
Serologic, molecular, and resistance-related variables				
Antibody titer score (0–5)	2.00 (0.00–5.00)a	4.00 (1.00–5.00)b	2.00 (0.00–5.00)a	0.004
A2063G resistance-site substitution, *n* (%)	143 (90.5)ab	115 (95.0)a	104 (84.6)b	0.023
Radiographic, management-related, and short-term outcome variables				
Pulmonary consolidation, *n* (%)	47 (29.7)	25 (20.7)	31 (25.2)	0.225
Bronchoalveolar lavage, *n* (%)	5 (3.2)	5 (4.1)	3 (2.4)	0.755
Intravenous corticosteroid use, *n* (%)	93 (58.9)	58 (47.9)	65 (52.8)	0.188
Supplemental oxygen use, *n* (%)	14 (8.9)	18 (14.9)	8 (6.5)	0.077
Oxygen therapy duration, days	2.0 (2.0–3.0)	3.0 (2.0–5.5)	3.5 (2.0–4.3)	0.434
PICU admission, *n* (%)	0 (0.0)	1 (0.8)	1 (0.8)	—
Mechanical ventilation, *n* (%)	0 (0.0)	1 (0.8)	0 (0.0)	—
Death, *n* (%)	0 (0.0)	0 (0.0)	0 (0.0)	—
Resource use				
Length of stay, days	7.00 (5.25–9.00)a	8.00 (6.00–10.00)b	7.00 (6.00–8.50)ab	0.028
Hospital cost, CNY	3,899.23 (3,023.50–4,661.03)a	4,256.35 (3,299.02–5,769.71)b	3,838.78 (3,224.99–5,201.76)a	0.033

Values are presented as median (IQR) or *n* (%). Letters following values indicate Bonferroni-adjusted pairwise differences; groups sharing at least one letter are not significantly different. The antibody titer score (0–5) represents ordinal categories of *Mycoplasma pneumoniae* antibody titers as defined in the Methods. Oxygen therapy duration was summarized among children who received supplemental oxygen. PICU admission, mechanical ventilation, and death were interpreted descriptively because events were rare or absent. IQR, interquartile range; Tmax, maximum body temperature; tNGS, targeted next-generation sequencing; MP, *Mycoplasma pneumoniae*; BMI, body mass index; WBC, white blood cell; N/L ratio, neutrophil-to-lymphocyte ratio; CRP, C-reactive protein; LDH, lactate dehydrogenase; BAL, bronchoalveolar lavage; PICU, pediatric intensive care unit; CNY, Chinese yuan.

Antibody titer scores were highest in the MP + viral co-detection group, indicating a relatively stronger serologic response in this subgroup. Radiographic and management-related variables, including pulmonary consolidation, bronchoalveolar lavage (BAL), and intravenous corticosteroid use, did not differ significantly across co-detection categories. Supplemental oxygen use was numerically higher in the MP + viral co-detection group, but the difference did not reach statistical significance. Oxygen therapy duration among oxygen users was also comparable across groups. Severe short-term outcomes were rare: two children required PICU admission, including one MP + viral co-detection case and one MP + bacterial co-detection case; the only mechanical ventilation event occurred in the MP + viral co-detection case. Both PICU cases had pulmonary consolidation and seronegative antibody titers at admission, with MP normalized read counts of 31,572 and 10,469, respectively, and both had prolonged hospitalization of 23 days. No deaths occurred in the full cohort. Length of stay and hospital costs were highest in the MP + viral co-detection group, suggesting slightly greater resource use.

In contrast, MP + bacterial co-detection generally resembled MP-only across most measured clinical, laboratory, radiographic, management-related, and short-term outcome variables, and did not show the same degree of clinical or inflammatory separation observed in the MP + viral co-detection group. The bacterial co-detection signals were mainly composed of common upper-airway respiratory bacteria, including Haemophilus influenzae, Streptococcus pneumoniae, Moraxella catarrhalis, Staphylococcus aureus, and streptococcal species; atypical panel organisms questioned for pediatric CAP relevance were rarely or not detected in the actual cohort.

The frequency of the A2063G substitution in the 23S rRNA gene was high in all three groups, with a slightly lower proportion in MP + bacterial co-detection than in MP-only and MP + viral co-detection. Without phenotypic susceptibility testing, this marker was interpreted primarily as describing the distribution of a resistance-associated locus rather than proven clinical non-response.

### Distribution patterns of antibody titers and normalized *Mycoplasma pneumoniae* reads

**Figure 4:**
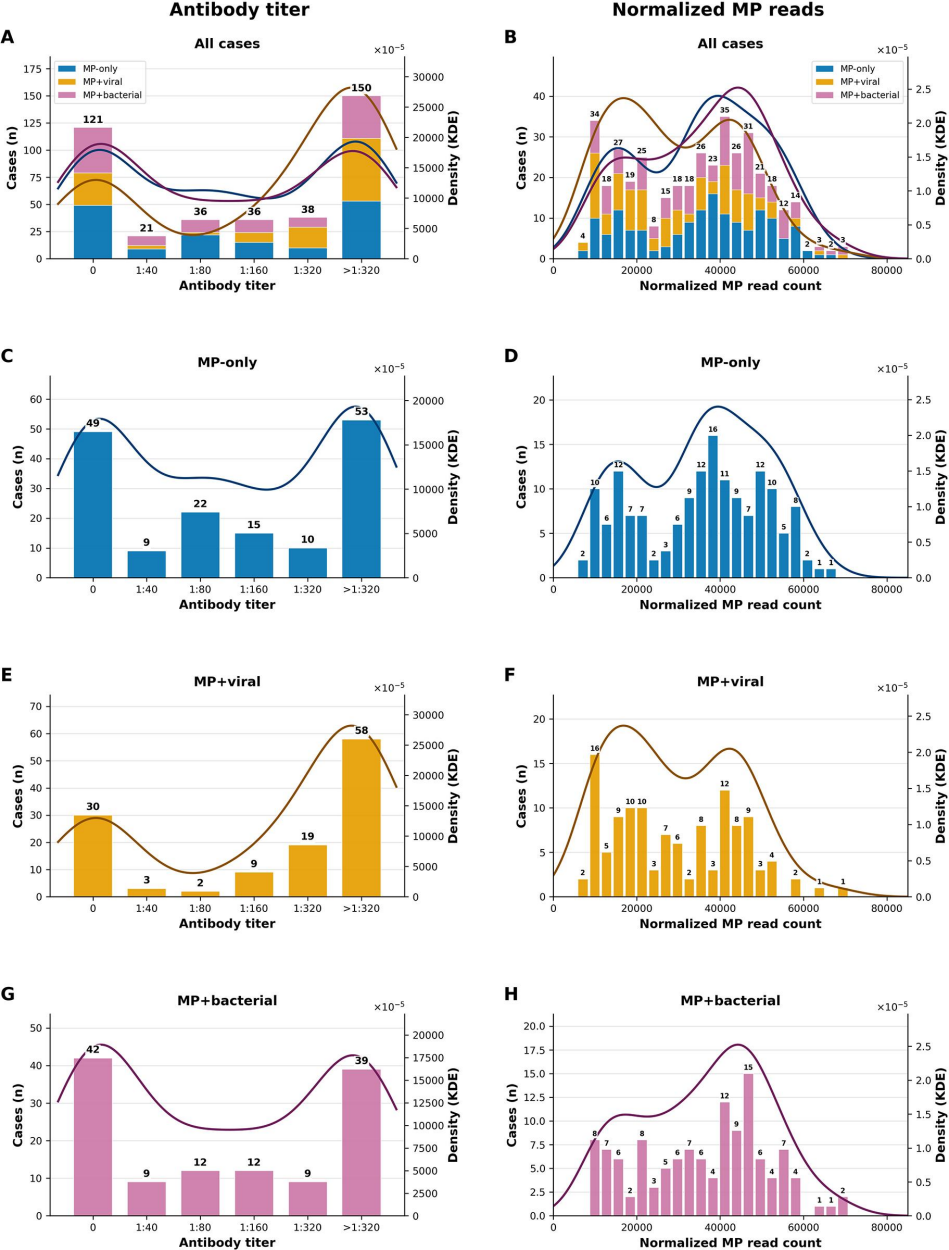
Distribution of antibody titers and normalized *Mycoplasma pneumoniae* reads across tNGS-based pathogen co-detection categories among hospitalized children. Panels **(A–H)** show histograms with overlaid kernel density estimates (KDEs) for antibody titers **(A,C,E,G)** and normalized *M. pneumoniae* reads **(B,D,F,H)**. Panel A displays cohort-level antibody titers for all 402 children, stacked by tNGS-based co-detection category, and panel **(B)** shows cohort-level normalized read counts. Panels **(C)** and **(D)** present titers and normalized reads, respectively, among children with MP-only pneumonia; panels **(E)** and **(F)** among children with MP + viral co-detection; and panels **(G)** and **(H)** among children with MP + bacterial co-detection.

At the cohort level, antibody titers showed a bimodal distribution, with peaks at seronegativity (tite*r* = 0) and very high titers (>1:320), and relatively fewer cases across intermediate dilutions (1:40–1:320) (Figure 4A). A similar pattern was observed within each co-detection category (Figures 4C–G), suggesting that many hospitalized children were captured either before measurable serologic response or after a strong humoral response had developed.

To further characterize the timing pattern of antibody emergence in this real-world cohort, onset-to-admission intervals were summarized across antibody titer categories (Supplementary Table S5). In the overall cohort, children with the first positive titer level of 1:40 were admitted at a median of 6 days after illness onset, and the same median interval was observed in the MP-only subgroup. Higher antibody titers corresponded to progressively longer onset-to-admission intervals, with the >1:320 group showing the longest interval. This cross-sectional timing gradient supports the interpretation that low-positive titers may represent an early seroconversion window, whereas very high titers more often reflect an established humoral response. Because antibody titers were measured only once at admission, these findings should be interpreted as population-level timing patterns rather than direct estimates of individual seroconversion time.

Normalized MP read distributions showed a visually apparent bimodal tendency, although the separation was less discrete than that observed for antibody titers (Figures 4B–H). At the cohort level, read counts clustered around a lower-read region and a higher-read region. The MP + viral co-detection group showed a more prominent lower-read peak, whereas the MP-only and MP + bacterial co-detection groups showed more prominent higher-read peaks. In the MP + bacterial co-detection group, the lower-read component was present but relatively flatter. Despite these distributional shifts, the read-count ranges overlapped substantially across co-detection categories; therefore, read-count distributions in co-detection categories were summarized descriptively. Formal load–titer phenotyping was prespecified in MP-only cases to reduce co-detection-related heterogeneity in normalized MP read interpretation. Because one-dimensional antibody titer or MP read distributions could not show how pathogen load and serologic response were paired within individual children, we next examined their joint distribution in MP-only cases using 2D-KDE.

### Admission-time molecular load–antibody titer patterns in children with MP-only pneumonia

**Figure 5:**
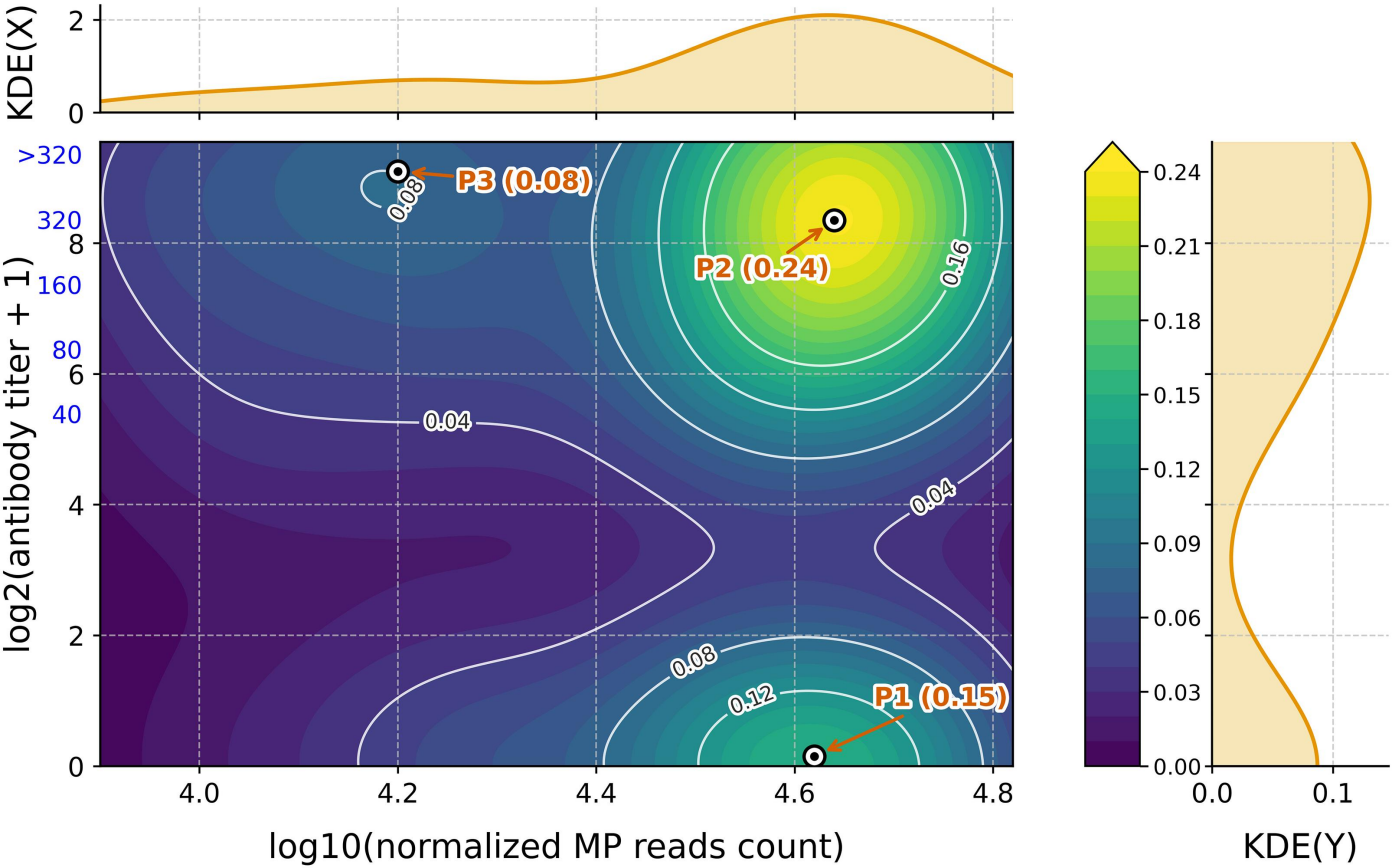
Joint distribution of tNGS-derived *Mycoplasma pneumoniae* molecular signal and antibody titers in children with MP-only pneumonia. Two-dimensional kernel density estimation (2D-KDE) of log10 (normalized *Mycoplasma pneumoniae* reads) and log2 (titer + 1) among 158 children with MP-only pneumonia. Color denotes joint density, with marginal KDEs for MP molecular signal (top) and antibody titers (right). Three density peaks define admission-time load–titer patterns: P1 high-load/seronegative, P2 high-load/high-titer, and P3 lower-load/high-titer. Arrows mark peak centers and contour lines indicate isodensity levels.

Within the MP-only subgroup (*n* = 158), the joint distribution of log₁₀(normalized MP reads) and log₂(titer + 1) showed a three-peak structure on 2D-KDE (Figure 5). Three data-driven admission-time load–titer patterns were defined: P1 high-load/seronegative, P2 high-load/high-titer, and P3 lower-load/high-titer. Using nearest-peak assignment in the transformed load–titer space, 47 (29.7%), 72 (45.6%), and 39 (24.7%) children with MP-only pneumonia were classified as P1, P2, and P3, respectively. Antibody titer scores and log₁₀(normalized MP reads) differed markedly across these patterns, confirming that P1–P3 represented distinct combinations of MP molecular signal and humoral response (Table 2).

**Table 2:** Clinical characteristics and short-term outcomes across admission-time load–titer patterns in children with MP-only pneumonia.

Variable	P1 high-load/seronegative (*n* = 47)	P2 high-load/high-titer (*n* = 72)	P3 lower-load/high-titer (*n* = 39)	*P* value
Demographics				
Age, months	60.00 (36.00–96.00)	66.00 (45.00–84.00)	72.00 (42.00–96.00)	0.953
Male sex, *n* (%)	31 (66.0)	33 (45.8)	23 (59.0)	0.083
BMI, kg/m²	14.58 (13.85–15.97)	14.74 (13.78–16.56)	14.50 (13.73–15.59)	0.819
Clinical course				
Fever duration, days	5.00 (4.00–5.00)a	6.00 (4.00–7.00)b	6.00 (5.00–7.00)b	<0.001
Tmax, °C	39.50 (38.95–40.00)	39.25 (38.80–39.50)	39.40 (39.00–39.60)	0.241
Onset-to-admission interval, days	5.00 (4.00–6.00)a	7.00 (5.00–8.00)b	7.00 (6.00–7.50)b	<0.001
Cough duration, days	4.00 (4.00–6.00)a	7.00 (5.00–7.00)b	6.00 (4.00–7.00)b	0.007
Laboratory markers				
WBC, ×10⁹/L	7.14 (6.01–9.24)	7.46 (6.18–9.27)	8.33 (7.07–9.51)	0.144
N/L ratio	1.72 (1.23–2.67)a	2.12 (1.54–2.92)ab	2.96 (1.56–4.38)b	0.018
Platelet count, ×10⁹/L	226.00 (183.50–283.00)a	284.00 (219.75–333.50)b	268.00 (227.00–313.00)ab	0.012
CRP, mg/L	17.70 (7.11–30.20)	12.07 (4.10–19.84)	15.30 (5.86–23.40)	0.216
LDH, U/L	318.00 (269.50–372.00)	322.50 (286.75–396.25)	334.00 (291.00–393.00)	0.570
Serologic and molecular markers				
Antibody titer score (0–5)	0.00 (0.00–0.00)a	4.00 (2.00–5.00)b	5.00 (2.00–5.00)b	<0.001
log₁₀(normalized MP reads)	4.57 (4.46–4.69)a	4.63 (4.58–4.71)b	4.16 (4.03–4.27)c	<0.001
A2063G resistance-site substitution, *n* (%)	45 (95.7)	63 (87.5)	35 (89.7)	0.319
Radiographic, management-related, and short-term outcome variables				
Pulmonary consolidation, *n* (%)	18 (38.3)	21 (29.2)	8 (20.5)	0.197
Bronchoalveolar lavage, *n* (%)	2 (4.3)	3 (4.2)	0 (0.0)	0.429
Intravenous corticosteroid use, *n* (%)	28 (59.6)	40 (55.6)	25 (64.1)	0.678
Supplemental oxygen use, *n* (%)	6 (12.8)	5 (6.9)	3 (7.7)	0.512
Oxygen therapy duration, days	2.0 (1.3–2.8)	2.0 (2.0–3.0)	3.0 (2.5–3.5)	0.556
PICU admission, *n* (%)	0 (0.0)	0 (0.0)	0 (0.0)	—
Mechanical ventilation, *n* (%)	0 (0.0)	0 (0.0)	0 (0.0)	—
Death, *n* (%)	0 (0.0)	0 (0.0)	0 (0.0)	—
Resource use				
Length of stay, days	8.00 (6.50–9.00)a	6.00 (5.00–8.25)b	6.00 (5.00–9.00)ab	0.039
Hospital cost, CNY	4,026.64 (3,425.37–4,667.59)	3,861.26 (3,005.34–4,630.35)	3,550.50 (2,852.14–4,685.14)	0.564

Values are presented as median (IQR) or *n* (%). Letters following values indicate Bonferroni-adjusted pairwise differences; groups sharing at least one letter are not significantly different. Oxygen therapy duration was summarized among children who received supplemental oxygen. PICU admission, mechanical ventilation, and death were interpreted descriptively because events were rare or absent. IQR, interquartile range; Tmax, maximum body temperature; P1, high-load/seronegative; P2, high-load/high-titer; P3, lower-load/high-titer; MP, *Mycoplasma pneumoniae*; BMI, body mass index; WBC, white blood cell; N/L ratio, neutrophil-to-lymphocyte ratio; CRP, C-reactive protein; LDH, lactate dehydrogenase; BAL, bronchoalveolar lavage; PICU, pediatric intensive care unit; CNY, Chinese yuan.

Clinical characteristics across the three admission-time load–titer patterns are summarized in Table 2. Across P1–P3, onset-to-admission interval, fever duration, and cough duration increased, supporting a timing-related interpretation. The N/L ratio also rose across the three patterns, and platelet counts peaked in P2 before partially declining in P3. In an age-adjusted sensitivity analysis using ln-transformed N/L ratio, the association between load–titer pattern and N/L ratio remained significant (overall P = 0.0066). The adjusted geometric mean N/L ratio increased from P1 to P3, with P3 remaining significantly higher than P1 after Bonferroni correction (Supplementary Table S4). These accompanying clinical and laboratory gradients suggest that the 3P structure was not merely a statistical partition of load and titer values, but was associated with interpretable timing and inflammatory features.

However, conventional admission severity proxies, including CRP, LDH, pulmonary consolidation, BAL, intravenous corticosteroid use, A2063G positivity, and hospital cost, did not clearly differ across P1–P3. Supplemental oxygen use and oxygen therapy duration among oxygen users also did not differ significantly across the three patterns. No PICU admission, mechanical ventilation, or death occurred in the MP-only subgroup. Length of stay showed only modest differences. Overall, the three-pattern structure was more consistent with timing-related molecular–serologic–inflammatory heterogeneity at admission than with sharply separated clinical severity categories.

In the threshold sensitivity analysis, 25 below-threshold children with low-signal MP-only pneumonia were added to the primary MP-only cohort, forming an expanded complete-case dataset of 183 children. Repeating the same log-transformed and standardized 2D-KDE procedure showed that the high-load/seronegative and high-load/high-titer regions corresponding to the primary P1 and P2 patterns remained identifiable. The added low-signal cases mainly extended the lower-load margin of the load–titer space, including very-low-load/high-titer and low-load/non–high-titer edge profiles. Because the low-load/seronegative accumulation included only five cases, this edge region was interpreted as exploratory and did not materially alter the interpretation of the primary P1–P3 load–titer patterns (Supplementary Table S3).

## Discussion

In this real-world cohort of hospitalized children with tNGS-confirmed MPP, we characterized two complementary aspects of admission-time interpretation. First, tNGS-based co-detection analysis in the full cohort showed that additional viral or bacterial signals were common in routine testing. Second, molecular load–antibody titer phenotyping in children with MP-only pneumonia revealed three dominant admission-time molecular–serologic patterns. These findings suggest that tNGS-derived MP molecular signal and antibody titers should be interpreted as complementary pathogen-side and host-side measurements rather than isolated binary results.

In the full cohort, MP-only pneumonia accounted for about two-fifths of children, while MP + viral and MP + bacterial co-detections each accounted for approximately one-third. These co-detection categories should be interpreted as upper-airway tNGS detection patterns rather than confirmed lower-respiratory coinfections. This distinction is particularly important for bacterial detections. Children with MP + bacterial co-detection generally resembled those with MP-only pneumonia across most measured clinical, laboratory, radiographic, management-related, and short-term outcome variables, and its monthly distribution was relatively even compared with the more seasonal increase observed for MP + viral co-detection. Together, these findings support cautious interpretation of bacterial reads from upper-airway specimens as possible colonization, background flora, low-abundance carriage, or non-causative co-detection rather than confirmed lower-respiratory bacterial pneumonia (12, 13). Therefore, bacterial co-detection should not automatically prompt etiologic attribution or treatment escalation without supportive clinical, inflammatory, and radiographic evidence.

Children with MP + viral co-detection showed a more distinct clinical profile, including younger age, longer cough duration, higher WBC and platelet counts, lower CRP, higher antibody titer scores, and slightly greater resource use. These findings are consistent with previous pediatric studies reporting that respiratory viruses are frequently detected in children hospitalized with MPP or community-acquired pneumonia and may modify clinical trajectories in selected cohorts (15–18). Rhinovirus was the most frequently detected virus and showed the broadest pairwise co-detection links, consistent with its central role in pediatric respiratory viral detection (15–21). Nevertheless, viral co-detection was also interpreted descriptively. Sporadic detections such as CMV should not be interpreted as evidence of viral pneumonia by themselves, and the viral co-detection network mainly illustrates pairwise detection patterns rather than causal interactions or proof of clinically meaningful viral coinfection.

A key observation was the bimodal distribution of antibody titers. Across the overall cohort and each co-detection category, titers clustered at seronegativity and at very high titers, with relatively fewer children at intermediate dilutions. This pattern is compatible with the known timing dependence of MP serology: some children are hospitalized before measurable antibody response has developed, whereas others are hospitalized after a strong humoral response is established (3, 8, 22, 23). However, serology alone cannot determine concurrent pathogen burden, and tNGS molecular signal alone cannot define the host immune response. This limitation motivated the MP-only load–titer analysis, in which the two measurements were examined jointly within a shared quantitative space.

To further clarify whether this bimodal titer distribution reflected illness timing, we summarized onset-to-admission intervals across antibody titer categories. In both the overall cohort and the MP-only subgroup, children with the first positive antibody titer level of 1:40 were typically admitted around day 6 after illness onset, whereas very high titers were associated with longer onset-to-admission intervals (Supplementary Table S5). This pattern is consistent with previous studies showing that MP serologic positivity depends strongly on the timing of sample collection and that antibody responses often become detectable around the first week of illness (8, 22, 23). Importantly, our finding does not define the exact seroconversion time in individual children, because titers were measured only once at admission. Rather, it provides a real-world, symptom-onset-based timing pattern showing that low-positive antibody titers cluster near the early humoral-response window in hospitalized children.

Within children with MP-only pneumonia, 2D-KDE identified three dominant admission-time molecular–serologic regions: P1 high-load/seronegative, P2 high-load/high-titer, and P3 lower-load/high-titer. These regions represent dominant admission-time patterns within a continuous molecular load–antibody titer space, rather than predefined clinical categories. Their formation is biologically plausible because antibody emergence, antibody persistence, and pathogen clearance are not synchronous, and hospitalization captures children at clinically selected time points rather than at uniform illness stages.

The most clinically informative finding was the P2 high-load/high-titer pattern. A simplified temporal interpretation might expect high MP molecular signal to coincide mainly with absent or low antibody titers and high antibody titers to coincide mainly with declining pathogen signal. Our results show that this interpretation is incomplete. In a substantial proportion of children with MP-only pneumonia, high MP molecular signal and established humoral response coexisted at admission. This high-load/high-titer state would be difficult to recognize using either tNGS signal or antibody titer alone and supports the added value of joint pathogen–host interpretation.

The accompanying clinical gradients supported a timing-related interpretation of P1–P3. Onset-to-admission interval, fever duration, and cough duration increased across the patterns. The N/L ratio also rose, and in an age-adjusted sensitivity analysis using ln-transformed N/L ratio, the association remained significant, with P3 higher than P1 after Bonferroni correction (Supplementary Table S4). Platelet counts peaked in P2 before partially declining in P3, suggesting that the high-load/high-titer region may coincide with a more active inflammatory phase. At the same time, conventional admission severity proxies, including CRP, LDH, pulmonary consolidation, BAL, corticosteroid use, A2063G positivity, and hospital cost, did not clearly separate P1–P3. Thus, these patterns appear to reflect molecular–serologic–inflammatory heterogeneity related to illness timing and host response rather than sharply stratified severity categories.

We also explored whether the admission-time load–titer patterns had short-term prognostic implications. Supplemental oxygen use and oxygen therapy duration did not differ significantly across P1–P3, and no PICU admission, mechanical ventilation, or death occurred in the MP-only subgroup. In the full cohort, severe adverse outcomes were uncommon, with only two PICU admissions, one mechanical ventilation event, and no deaths. Notably, the two PICU cases did not share an extremely high MP molecular signal; one had a moderate MP normalized read count and the other had a lower-range read count, while both were seronegative at admission and had pulmonary consolidation. These observations do not support MP molecular load or admission-time seronegativity as stand-alone prognostic markers in this general hospitalized cohort. Because antibody titers were measured only once at admission, P1 should be interpreted as an admission-time seronegative pattern rather than sustained antibody non-response. Whether load–titer patterns predict adverse outcomes requires larger cohorts enriched for severe or refractory MPP and with longitudinal sampling.

The threshold sensitivity analysis further clarified the boundary of this interpretation. After adding below-threshold low-signal children with MP-only pneumonia and repeating the same 2D-KDE procedure, the high-load/seronegative and high-load/high-titer regions corresponding to primary P1 and P2 remained identifiable. The added low-signal children mainly extended the lower-load margin of the load–titer space, including very-low-load/high-titer and low-load/non–high-titer edge profiles. The very-low-load/high-titer children are compatible with antibody persistence after MP molecular signal declines, whereas the low-load/seronegative accumulation included only five children and was insufficient to define a formal additional phenotype. These findings support treating below-threshold children as exploratory low-signal margins rather than redefining the primary P1–P3 structure.

From a testing-strategy perspective, the added value of antibody titers in the tNGS era is not to duplicate MP detection, but to help interpret the clinical meaning of an MP-positive tNGS result. This may be particularly relevant in children with mixed-pathogen detections and low MP molecular signal. Around one week after illness onset, persistently negative antibody titers should prompt cautious interpretation of whether MP represents the main current pathogen, especially when another detected respiratory pathogen better explains the clinical presentation. Conversely, a low-positive antibody titer around this early window may support that the MP molecular signal is occurring in the context of an emerging immune response. Similarly, in children with high MP molecular signal, antibody titers may help distinguish an early high-load/seronegative state from a high-load/high-titer state in which persistent MP molecular signal and established humoral response coexist. These interpretations should not dictate treatment automatically, but they may help clinicians contextualize MP tNGS results in relation to illness timing, host immune response, and the full co-detection profile.

Several methodological points require caution. Normalized tNGS reads are semi-quantitative and platform-dependent. Although metagenomic or next-generation sequencing readouts normalized as reads per million (RPM) or similar metrics may correlate with quantitative polymerase chain reaction or cycle threshold (Ct)-derived abundance, they remain sensitive to specimen type, sampling conditions, and compositional effects (24–26). Therefore, we interpreted log₁₀(normalized reads) as a within-cohort proxy of upper-airway MP molecular signal rather than an absolute pathogen load. This differs from BALF-based or high-risk/refractory cohorts, where higher lower-airway MP DNA loads have been more consistently associated with severe or refractory disease (24–27). In addition, the high prevalence of the 23S rRNA A2063G substitution is consistent with the circulation of macrolide-resistant MP in East Asia (5, 14, 19, 20). A2063G positivity exceeded 80% in all co-detection categories and was highest in MP + viral co-detection, lowest in MP + bacterial co-detection, and intermediate in MP-only. Without phenotypic susceptibility testing, this marker should be interpreted as a resistance-associated locus rather than direct evidence of clinical macrolide non-response.

This study has limitations. It was a single-center retrospective study of hospitalized children, and the findings may not generalize to outpatient MPP, mild disease, or other geographic settings. All tNGS specimens were oropharyngeal swabs, limiting etiologic inference for bacterial detections and lower-airway pathogen burden. Normalized read counts are semi-quantitative, and the absolute thresholds and density patterns require external validation. The estimated MP concentration threshold may have excluded low-signal MP-positive children, although sensitivity analysis suggested that these children mainly expanded the low-load margin. Finally, P1–P3 were derived from admission-time cross-sectional data and should not be interpreted as proven longitudinal stages. Because only admission-time antibody titers were available and severe outcomes were infrequent, sustained antibody responses and prognostic implications could not be fully evaluated. Serial sampling and larger severe-case-enriched cohorts are needed to confirm transitions between patterns and to test whether these patterns predict treatment response or long-term outcomes.

In summary, pathogen co-detection was common among hospitalized children with MPP and should be interpreted according to specimen type and clinical context. In children with MP-only pneumonia, admission-time molecular load–antibody titer phenotyping suggested three dominant immunologic patterns, including a high-load/high-titer state in which persistent MP molecular signal and established humoral response coexist. This joint framework may help clinicians interpret pathogen burden in relation to host immune status and illness timing more appropriately than relying on molecular load or antibody titer alone.

## Data Availability

The datasets generated and/or analysed during the current study are not publicly available due to patient privacy and institutional regulations, but de-identified data may be made available from the corresponding author on reasonable request and with permission from the Ethics Committee of Shantou Central Hospital.
