# Supplementary material for: Admission-time immunologic patterns in hospitalized children with Mycoplasma pneumoniae pneumonia: a molecular load–antibody titer phenotyping analysis
Source: Front Pediatr. 2026 Jul 15;14:1814508. doi: 10.3389/fped.2026.1814508 (PMC13416547; doi:10.3389/fped.2026.1814508)
Supplement: Supplementary file 1 [file Datasheet1.docx]

**Supplementary Methods**

*Semi-quantitative normalization of tNGS read counts*

To strengthen interpretability of log₁₀(RPK), we applied the following constraints:

1. Depth normalization and specimen restriction: normalization to non-host reads; restriction to oropharyngeal swabs collected under a standardized procedure; and restriction to MP-only cases for load–titer distribution analyses to avoid potential denominator distortion from additional pathogens;

2. Standardized laboratory workflow: all specimens were processed in a single laboratory using the same platform, reagent panel, and internal QC criteria;

3. Restriction to high-load samples: inclusion required estimated MP concentration >1 × 10⁶ copies/mL to reduce stochastic noise near the detection limit and to focus analyses on the assay’s high-load range.

Under these conditions, log₁₀(RPK) was interpreted as a semi-quantitative indicator of MP nucleic acid signal and used for distributional/pattern analyses rather than absolute quantification.

*Antibody titer handling*

Titers were analyzed in two complementary ways:

1. Ordinal titer score (0–5) for group comparisons (Tables 1 and 3): 0 = negative, 1 = 1:40, 2 = 1:80, 3 = 1:160, 4 = 1:320, 5 = >1:320;

2. Semi-quantitative continuous metric for visualization and correlation analyses: titers were mapped to numeric values (0, 40, 80, 160, 320, and 640 for >1:320) and transformed as log₂(titer + 1). This variable was used as the vertical axis in 2D-KDE plots and in correlation analyses with log₁₀(RPK) (Figure 5).
