# Supplementary material for: Admission-time immunologic patterns in hospitalized children with Mycoplasma pneumoniae pneumonia: a molecular load–antibody titer phenotyping analysis
Source: Front Pediatr. 2026 Jul 15;14:1814508. doi: 10.3389/fped.2026.1814508 (PMC13416547; doi:10.3389/fped.2026.1814508)
Supplement: Supplementary file 2 [file Table1.docx]

**Supplementary Table S1. Pathogens detected by targeted next-generation sequencing (tNGS)**

| Category | Detected pathogens |
| --- | --- |
| Gram-positive bacteria | Streptococcus pneumoniae, Streptococcus pyogenes, Streptococcus agalactiae, Listeria monocytogenes, Streptococcus anginosus group, Streptococcus intermedius, Streptococcus dysgalactiae, Corynebacterium ulcerans, Staphylococcus aureus, Corynebacterium diphtheriae, Arcanobacterium haemolyticum |
| Gram-negative bacteria | Haemophilus influenzae, Bordetella pertussis, Moraxella catarrhalis, Neisseria meningitidis, Yersinia enterocolitica, Fusobacterium necrophorum, Legionella pneumophila, Neisseria gonorrhoeae |
| DNA viruses | Herpes simplex virus type 1 (HSV-1), Herpes simplex virus type 2 (HSV-2), Human herpesvirus 6 (HHV-6), Human herpesvirus 7 (HHV-7), Epstein–Barr virus (EBV), Cytomegalovirus (CMV), Varicella–zoster virus (VZV), Human parvovirus B19, Human bocavirus type 1 (HBoV-1), Human adenovirus (HAdV) |
| RNA viruses | Human parainfluenza virus types 1–4 (HPIV-1/2/3/4), Human metapneumovirus (HMPV), Influenza A virus, Influenza B virus, Influenza C virus, Respiratory syncytial virus A/B (RSV-A/B), Enterovirus, Rhinovirus, Measles virus, Rubella virus, Mumps virus, SARS-CoV-2, Human coronavirus HKU1, NL63, OC43, 229E |
| Mycoplasma species | Mycoplasma pneumoniae, Ureaplasma urealyticum, Mycoplasma hominis, Ureaplasma parvum, Mycoplasma genitalium |
| Chlamydia species | Chlamydia psittaci, Chlamydia pneumoniae, Chlamydia trachomatis |

Note: This table lists the full detectable pathogen spectrum of the tNGS panel used in this study, not the pathogens actually detected in the cohort. Actual co-detection profiles are summarized in the Results. Bacterial signals from upper-airway specimens were interpreted as co-detections rather than confirmed lower-respiratory bacterial coinfections.
