# Supplementary material for: Admission-time immunologic patterns in hospitalized children with Mycoplasma pneumoniae pneumonia: a molecular load–antibody titer phenotyping analysis
Source: Front Pediatr. 2026 Jul 15;14:1814508. doi: 10.3389/fped.2026.1814508 (PMC13416547; doi:10.3389/fped.2026.1814508)
Supplement: Supplementary file 3 [file Table2.docx]

**Supplementary Table S2. Four-group sensitivity analysis of tNGS-based pathogen co-detection categories**

A. Clinical characteristics across four tNGS-based pathogen co-detection categories

| Variable | MP-only  (n=158) | MP+viral only  (n=59) | MP+bacterial only  (n=123) | MP+viral+bacterial co-detection (n=62) | P value |
| --- | --- | --- | --- | --- | --- |
| **Demographics** |  |  |  |  |  |
| Age, months | 66.0 (36.0–96.0) | 36.0 (18.0–72.0) | 60.0 (36.0–84.0) | 36.0 (12.0–60.0) | <0.001 |
| Male sex, n (%) | 87 (55.1) | 34 (57.6) | 71 (57.7) | 38 (61.3) | 0.865 |
| BMI | 14.58 (13.80–16.06) | 14.79 (13.79–16.01) | 14.61 (13.69–15.82) | 15.14 (13.81–16.27) | 0.652 |
| **Clinical course** |  |  |  |  |  |
| Fever duration before admission, days | 5.0 (4.0–7.0) | 5.0 (2.0–7.0) | 5.0 (3.0–6.5) | 3.5 (1.2–6.0) | 0.017 |
| Tmax, °C | 39.3 (38.9–39.7) | 39.0 (38.2–39.5) | 39.3 (38.7–39.8) | 39.0 (38.5–39.4) | 0.010 |
| Onset-to-admission interval, days | 7.00 (5.00–7.00) | 7.00 (5.00–10.00) | 6.00 (5.00–8.50) | 7.00 (4.25–10.00) | 0.078 |
| Cough duration at admission, days | 6.00 (4.00–7.00) | 7.00 (5.00–10.00) | 5.00 (4.00–8.00) | 7.00 (4.00–10.00) | 0.030 |
| **Laboratory markers** |  |  |  |  |  |
| WBC, ×10⁹/L | 7.7 (6.3–9.2) | 9.0 (7.0–10.9) | 8.2 (6.3–10.6) | 9.3 (6.8–11.2) | 0.015 |
| N/L ratio | 2.13 (1.46–3.23) | 1.87 (1.25–2.77) | 2.01 (1.51–3.00) | 1.78 (1.06–2.41) | 0.156 |
| Platelet count, ×10⁹/L | 263.0 (213.2–319.5) | 294.0 (223.0–379.0) | 250.0 (213.0–323.5) | 310.0 (227.0–393.8) | 0.011 |
| CRP, mg/L | 13.7 (4.4–24.6) | 8.34 (0.00–19.20) | 12.0 (4.2–25.3) | 7.5 (0.1–13.7) | 0.013 |
| LDH, U/L | 324.5 (285.0–391.8) | 337.0 (288.0–410.0) | 329.0 (277.0–375.5) | 334.0 (289.5–394.0) | 0.374 |
| **Serologic and resistance marker** |  |  |  |  |  |
| Antibody titer score (0–5) | 2.00 (0.00–5.00) | 5.00 (3.00–5.00) | 2.00 (0.00–5.00) | 4.00 (0.00–5.00) | 0.002 |
| A2063G resistance-site substitution, n (%) | 143 (90.5) | 56 (94.9) | 104 (84.6) | 59 (95.2) | 0.056 |
| **Radiographic and management variables** |  |  |  |  |  |
| Pulmonary consolidation, n (%) | 47 (29.7) | 12 (20.3) | 31 (25.2) | 13 (21.0) | 0.393 |
| BAL, n (%) | 5 (3.2) | 3 (5.1) | 3 (2.4) | 2 (3.2) | 0.826 |
| Intravenous corticosteroid use, n (%) | 93 (58.9) | 28 (47.5) | 65 (52.8) | 30 (48.4) | 0.340 |
| **Resource use** |  |  |  |  |  |
| Length of stay, days | 7.00 (5.25–9.00) | 8.00 (6.00–10.00) | 7.00 (6.00–8.50) | 8.00 (6.00–10.00) | 0.053 |
| Hospital cost, CNY | 3899.23 (3023.50–4661.03) | 4256.35 (3299.02–5769.71) | 3838.78 (3224.99–5201.76) | 4414.27 (3450.37–5758.05) | 0.054 |

**Note:** This four-group table separates MP+viral only and MP+viral+bacterial co-detection to evaluate the robustness of the primary three-category grouping strategy. Values are presented as median (IQR) or n (%). P values were calculated using the Kruskal–Wallis test for continuous or ordinal variables and the chi-square test or Fisher’s exact test for categorical variables, as appropriate. Categories describe tNGS-based co-detection results and are not intended to establish causality or confirm lower-respiratory tract coinfection.

B. Pairwise comparison between MP+viral only and MP+viral+bacterial co-detection groups

| Variable | P value |
| --- | --- |
| **Demographics** |  |
| Age, months | 0.551 |
| Male sex, n (%) | 0.822 |
| BMI | 0.283 |
| **Clinical course** |  |
| Fever duration before admission, days | 0.022 |
| Tmax, °C | 0.521 |
| Onset-to-admission interval, days | 0.409 |
| Cough duration at admission, days | 0.451 |
| **Laboratory markers** |  |
| WBC, ×10⁹/L | 0.895 |
| N/L ratio | 0.122 |
| Platelet count, ×10⁹/L | 0.251 |
| CRP, mg/L | 0.147 |
| LDH, U/L | 0.635 |
| **Serologic and resistance marker** |  |
| Antibody titer score (0–5) | 0.039 |
| A2063G resistance-site substitution, n (%) | 1.000 |
| **Radiographic and management variables** |  |
| Pulmonary consolidation, n (%) | 1.000 |
| BAL, n (%) | 0.674 |
| Intravenous corticosteroid use, n (%) | 1.000 |
| **Resource use** |  |
| Length of stay, days | 0.516 |
| Hospital cost, CNY | 0.438 |

Note: The pairwise comparison was performed to assess whether MP+viral+bacterial co-detection should be analyzed separately from MP+viral only co-detection. Continuous or ordinal variables were compared using the Mann–Whitney U test, and categorical variables were compared using the chi-square test or Fisher’s exact test, as appropriate. The antibody titer score (0–5) represents ordinal categories of M. pneumoniae antibody titers as defined in the Methods. MP, Mycoplasma pneumoniae; tNGS, targeted next-generation sequencing; N/L ratio, neutrophil-to-lymphocyte ratio; CRP, C-reactive protein; LDH, lactate dehydrogenase; BAL, bronchoalveolar lavage.

Summary of the sensitivity analysis: MP+viral only and MP+viral+bacterial co-detection showed broadly similar clinical, inflammatory, radiographic, management-related, and resource-use profiles, with differences mainly limited to fever duration and antibody titer score. These findings supported merging these two categories into a single MP+viral co-detection group for the primary analysis.
