# Supplementary material for: Admission-time immunologic patterns in hospitalized children with Mycoplasma pneumoniae pneumonia: a molecular load–antibody titer phenotyping analysis
Source: Front Pediatr. 2026 Jul 15;14:1814508. doi: 10.3389/fped.2026.1814508 (PMC13416547; doi:10.3389/fped.2026.1814508)
Supplement: Supplementary file 4 [file Table3.docx]

**Supplementary Table S3. Threshold sensitivity analysis after adding low-signal MP-only cases with available antibody titers**

A. Repeated 2D-KDE structure after adding low-signal MP-only cases

| Load–titer region | Primary MP-only cohort (n = 158) | Expanded MP-only complete-case cohort (n = 183) |
| --- | --- | --- |
| High-load / seronegative | P1 identified; peak at log₁₀(reads)=4.618, log₂(titer+1)=0.000 | Corresponding region remained identifiable; peak at log₁₀(reads)=4.577, log₂(titer+1)=0.015 (n=0) |
| High-load / high-titer | P2 identified; peak at log₁₀(reads)=4.645, log₂(titer+1)=8.355 | Corresponding region remained identifiable; peak at log₁₀(reads)=4.600, log₂(titer+1)=8.384 (n=0) |
| Lower-load / high-titer | P3 identified; peak at log₁₀(reads)=4.187, log₂(titer+1)=8.842 | Lower-load/high-titer margin was extended; very-low-load/high-titer edge at log₁₀(reads)=1.928, log₂(titer+1)=8.296 (n=9) |
| Low-load / low-titer edge | Not defined as a primary phenotype | Low-load/ seronegative and low-intermediate-titer edge profiles were observed (n=16) |

Note:Values in parentheses in Part A indicate the number of added low-signal cases contributing to the corresponding region, not the total number of cases in that region.

B. Comparison of primary P1–P3 patterns and low-signal MP-only cases

| Variable | P1 high-load/seronegative (n=47) | P2 high-load/high-titer (n=72) | P3 lower-load/high-titer (n=39) | Low-signal MP-only cases (n=25) | P value |
| --- | --- | --- | --- | --- | --- |
| Age, months | 60 (36–96) | 66 (45–84) | 72 (42–96) | 84 (60–120) | 0.226 |
| Male sex, n (%) | 31 (66.0) | 33 (45.8) | 23 (59.0) | 8 (32.0) | 0.025 |
| Fever duration before admission, days | 5.00 (4.00–5.00) | 6.00 (4.00–7.00) | 6.00 (5.00–7.00) | 6.00 (4.00–7.00) | 0.002 |
| Tmax, °C | 39.50 (38.95–40.00) | 39.25 (38.80–39.50) | 39.40 (39.00–39.60) | 39.50 (39.00–39.60) | 0.357 |
| Onset-to-admission interval, days | 5.00 (4.00–6.00) | 7.00 (5.00–8.00) | 7.00 (6.00–7.50) | 6.00 (4.00–7.00) | <0.001 |
| Cough duration at admission, days | 4.00 (4.00–6.00) | 7.00 (5.00–7.00) | 6.00 (4.00–7.00) | 6.00 (5.00–7.00) | 0.009 |
| Antibody titer score (0–5) | 0.00 (0.00–0.00) | 4.00 (2.00–5.00) | 5.00 (2.00–5.00) | 3.00 (1.00–5.00) | <0.001 |
| Seronegative antibody titer, n (%) | 47 (100.0) | 0 (0.0) | 2 (5.1) | 5 (20.0) | <0.001 |
| Very high antibody titer (>1:320), n (%) | 0 (0.0) | 33 (45.8) | 20 (51.3) | 9 (36.0) | <0.001 |
| log₁₀(normalized MP reads) | 4.57 (4.46–4.69) | 4.63 (4.58–4.71) | 4.16 (4.03–4.27) | 3.32 (2.84–3.56) | <0.001 |
| WBC, ×10⁹/L | 7.14 (6.01–9.24) | 7.46 (6.18–9.27) | 8.33 (7.07–9.51) | 7.49 (6.40–9.42) | 0.263 |
| N/L ratio | 1.72 (1.23–2.67) | 2.12 (1.54–2.92) | 2.96 (1.56–4.38) | 2.51 (1.87–4.28) | 0.007 |
| Platelet count, ×10⁹/L | 226.00 (183.50–283.00) | 284.00 (219.75–333.50) | 268.00 (227.00–313.00) | 255.00 (214.00–325.00) | 0.029 |
| CRP, mg/L | 17.70 (7.11–30.20) | 12.07 (4.10–19.84) | 15.30 (5.86–23.40) | 18.70 (7.22–29.43) | 0.251 |
| LDH, U/L | 318.00 (269.50–372.00) | 322.50 (286.75–396.25) | 334.00 (291.00–393.00) | 336.00 (301.00–389.00) | 0.602 |
| Pulmonary consolidation, n (%) | 18 (38.3) | 21 (29.2) | 8 (20.5) | 10 (40.0) | 0.232 |
| BAL, n (%) | 2 (4.3) | 3 (4.2) | 0 (0.0) | 0 (0.0) | 0.609 |
| Intravenous corticosteroid use, n (%) | 28 (59.6) | 40 (55.6) | 25 (64.1) | 15 (60.0) | 0.850 |
| Length of stay, days | 8.00 (6.50–9.00) | 6.00 (5.00–8.25) | 6.00 (5.00–9.00) | 6.00 (5.00–8.00) | 0.036 |
| Hospital cost, CNY | 4026.64 (3425.37–4667.59) | 3861.26 (3005.34–4630.35) | 3550.50 (2852.14–4685.14) | 4121.76 (3177.11–4602.81) | 0.784 |

Note: The expanded MP-only complete-case cohort comprised the primary MP-only cohort (n = 158) plus low-signal MP-only cases with available antibody titers (n = 25). The same log-transformed and standardized 2D-KDE procedure was repeated in the expanded dataset. Values in parentheses in Part A indicate the number of added low-signal cases contributing to the corresponding region, not the total number of cases in that region. Part B compares the low-signal cases with the primary P1–P3 patterns; P values are exploratory and were calculated using the Kruskal–Wallis test for continuous or ordinal variables and Fisher’s exact test for categorical variables. Values are presented as median (IQR), n (%), or range as indicated.
