# Supplementary material for: Admission-time immunologic patterns in hospitalized children with Mycoplasma pneumoniae pneumonia: a molecular load–antibody titer phenotyping analysis
Source: Front Pediatr. 2026 Jul 15;14:1814508. doi: 10.3389/fped.2026.1814508 (PMC13416547; doi:10.3389/fped.2026.1814508)
Supplement: Supplementary file 5 [file Table4.docx]

**Supplementary Table S4. Age-adjusted analysis of N/L ratio across admission-time load–titer patterns in MP-only cases**

A. Age-adjusted geometric mean N/L ratio

| **Measure** | **P1 high-load/seronegative (n=47)** | **P2 high-load/high-titer (n=72)** | **P3 lower-load/high-titer (n=39)** | **Overall P value** |
| --- | --- | --- | --- | --- |
| Adjusted geometric mean N/L ratio (95% CI) | 1.78 (1.52–2.09) | 2.00 (1.76–2.28) | 2.60 (2.18–3.09) | 0.0066 |

B. Pairwise comparisons of adjusted geometric means

| **Comparison** | **Ratio of adjusted geometric means** | **Bonferroni-adjusted P value** |
| --- | --- | --- |
| P2 vs P1 | 1.12 | 0.782 |
| P3 vs P1 | 1.46 | 0.0057 |
| P3 vs P2 | 1.30 | 0.0556 |

Note: Age-adjusted differences in N/L ratio across P1–P3 were assessed using a linear model with ln-transformed N/L ratio as the dependent variable, load–titer pattern as the main factor, and age in months as a covariate. Adjusted geometric means were obtained by back-transforming model-estimated marginal means. Pairwise comparisons were adjusted using the Bonferroni method. N/L ratio, neutrophil-to-lymphocyte ratio.
