# Supplementary material for: Admission-time immunologic patterns in hospitalized children with Mycoplasma pneumoniae pneumonia: a molecular load–antibody titer phenotyping analysis
Source: Front Pediatr. 2026 Jul 15;14:1814508. doi: 10.3389/fped.2026.1814508 (PMC13416547; doi:10.3389/fped.2026.1814508)
Supplement: Supplementary file 6 [file Table5.docx]

**Supplementary Table S5. Onset-to-admission interval across antibody titer categories in the overall cohort and MP-only subgroup**

| Antibody titer group | Overall n | Overall onset-to-admission interval, days | MP-only n | MP-only onset-to-admission interval, days |
| --- | --- | --- | --- | --- |
| 0 | 121 | 5 (4–7) | 49 | 5 (4–6) |
| 1:40 | 21 | 6 (5–7) | 9 | 6 (5–7) |
| 1:80 | 36 | 6 (5–7) | 22 | 6 (5.5–7) |
| 1:160 | 36 | 6 (5–7) | 15 | 6 (5–7) |
| 1:320 | 38 | 7 (5–8) | 10 | 7 (7–7.5) |
| >1:320 | 150 | 8 (7–10) | 53 | 7 (7–9) |

Note: Values are presented as median (IQR). Antibody titers were measured once at admission; therefore, these data describe cross-sectional timing patterns rather than individual seroconversion time. Kruskal–Wallis tests showed significant differences in onset-to-admission interval across antibody titer categories in both the overall cohort and the MP-only subgroup (both P < 0.001). MP, *Mycoplasma pneumoniae*.
